# Evaluation of Trends in Alcohol Use Disorder–Related Mortality in the US Before and During the COVID-19 Pandemic

**DOI:** 10.1001/jamanetworkopen.2022.10259

**Published:** 2022-05-04

**Authors:** Yee Hui Yeo, Xinyuan He, Peng-Sheng Ting, Jian Zu, Christopher V. Almario, Brennan M. R. Spiegel, Fanpu Ji

**Affiliations:** 1 Division of General Internal Medicine, Cedars-Sinai Medical Center, Los Angeles, California; 2 Department of Infectious Diseases, Second Affiliated Hospital of Xi’an Jiaotong University, Xi’an, Shaanxi, China; 3 Division of Gastroenterology and Hepatology, Department of Medicine, Johns Hopkins University School of Medicine, Baltimore, Maryland; 4 School of Mathematics and Statistics, Xi’an Jiaotong University, Xi’an, Shaanxi, China; 5 Karsh Division of Gastroenterology and Hepatology, Cedars-Sinai Medical Center, Los Angeles, California

## Abstract

This cross-sectional study uses US vital statistics data to evaluate alcohol use disorder–related mortality rates from 2012 to 2021, with a focus on trends during the COVID-19 pandemic.

## Introduction

The US mortality rate has surged during the COVID-19 pandemic. Therefore, it is imperative to identify diseases and health conditions that have been affected disproportionately. Mounting evidence indicates that alcohol sales, alcohol consumption, and complications of alcohol use have increased during the pandemic.^1,2,3^ However, there are limited data on nationwide alcohol use disorder (AUD)–related mortality. Here, we use projective modeling to evaluate AUD-related mortality rates in the US from 2012 to 2021, with a focus on trends during the COVID-19 pandemic.

## Methods

This cross-sectional study used deidentified publicly available data, so informed consent and institutional review board approval were not required in accordance with the Common Rule. The study followed the STROBE reporting guideline.

We obtained deidentified data from the National Vital Statistics System (NVSS) through the Centers for Disease Control and Prevention Wide-Ranging Online Data for Epidemiologic Research (CDC WONDER) database.^4^ The NVSS database registers more than 99% of deaths in the US, and this study used data updated to January 22, 2022.^5^ We included decedents with AUD, defined by *International Classification of Diseases, Tenth Revision* diagnosis codes, as one cause of death (multiple causes were possible). Age-standardized mortality rates were estimated with the indirect method using the 2000 US Census as the standard population. We performed linear regression analysis to determine 2020 and 2021 projected mortality rates based on trends between 2012 and 2019. We quantified the association of the pandemic with AUD-related deaths by calculating percentage differences between the projected and observed mortality rates. To enrich robustness, we also performed a sensitivity analysis by setting AUD as the underlying (primary) cause of death. Statistical analyses were performed using the CDC WONDER database (age standardization), R version 4.0.2 (data cleaning and management), and PyCharm version 3.9.0 (modeling analysis).

## Results

There were 343 384 AUD-related deaths between 2012 and 2021. We stratified these deaths into decedent groups by age (25 to 44 years, 56 985 [16.6%]; 45 to 64 years, 192 346 [56.0%]; and ≥65 years, 94 053 [27.4%]) and by sex (266 755 men [77.7%]). By comparing observed and projected mortality rates, we noticed a surge in AUD mortality both overall and among all subgroups during the pandemic (Figure and Table). The observed AUD-related mortality rates increased by 24.79% in 2020 and 21.95% in 2021 vs the projected rates. Increased mortality rates were evident even when AUD was set as the underlying cause of death (30.74% in 2020 vs 28.77% in 2021).

**Figure.**
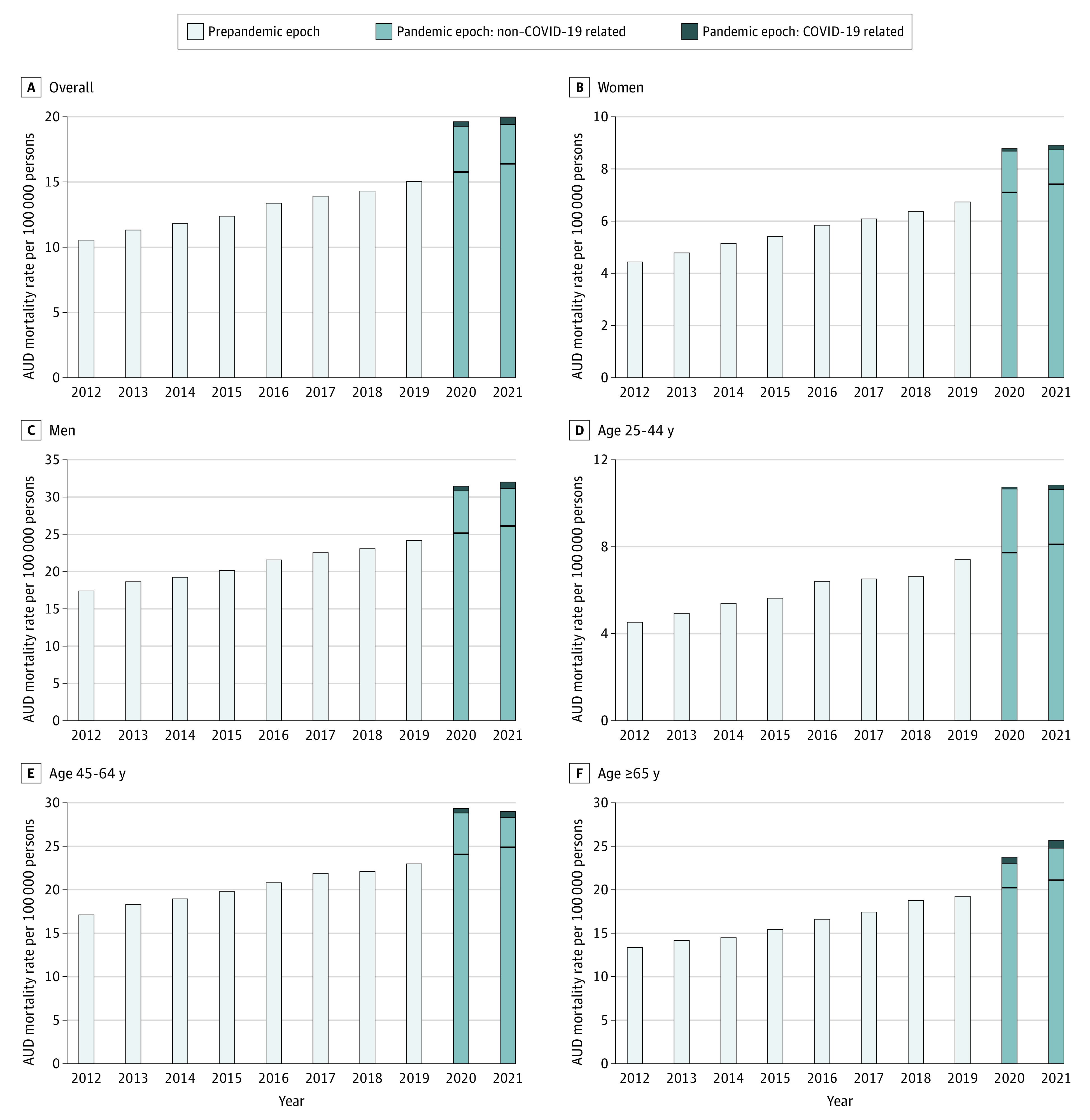
Trends in Alcohol Use Disorder–Related Mortality Before and During the COVID-19 Pandemic, 2012 to 2021 Mortality rates are presented for decedent groups stratified by sex and age. Linear regression was used to obtain projected values according to trends from 2012 to 2019. Horizontal black lines denote projected mortality rates.

**Table.** Age-Standardized and Projected Mortality Rates of Alcohol Use Disorder–Related Mortality Stratified by Decedent Age Group and Sex, 2012 to 2021

Variable	Age-standardized mortality rate per 100 000 persons							
	2012 Observed	2019 Observed	2020			2021		
			Observed	Projected (95% CI)	% Difference^a^	Observed	Projected (95% CI)	% Difference^a^
AUD-related mortality								
Multiple causes of death	10.63	15.11	19.68	15.77 (15.38-16.15)	+24.79	20.00	16.40 (15.99-16.82)	+21.95
Multiple causes of death (non–COVID-19)^b^	10.63	15.11	19.34	15.77 (15.38-16.15)	+22.64	19.48	16.40 (15.99-16.82)	+18.78
Underlying cause of death^c^	3.05	4.57	6.21	4.75 (4.58-4.92)	+30.74	6.40	4.97 (4.78-5.15)	+28.77
Age, y								
25-44	4.55	7.43	10.83	7.71 (7.17-8.25)	+40.47	10.85	8.10 (7.52-8.68)	+33.95
45-64	17.14	23.00	29.21	24.01 (23.28-24.74)	+21.66	28.95	24.84 (24.06-25.62)	+16.55
≥65	13.53	19.40	23.83	20.31 (19.48-21.14)	+17.33	25.83	21.19 (20.30-22.08)	+21.90
Sex								
Women	4.47	6.77	8.85	7.10 (6.96-7.23)	+24.65	8.91	7.42 (7.28-7.57)	+20.08
Men	17.37	24.15	31.35	25.15 (24.48-25.83)	+24.65	31.96	26.11 (25.39-26.84)	+22.41
Referent								
All-cause mortality	1097.84	1071.78	1254.44	1077.69 (1056.87-1098.50)	+16.40	1271.54	1074.99 (1052.68-1097.30)	+18.28

Abbreviation: AUD, alcohol use disorder.

^a^ Percentage differences were calculated as follows: (Observed – Projected)/Projected × 100%.

^b^ Excluded decedents with COVID-19 infection as the underlying cause of death.

^c^ Included decedents with AUD listed as the primary cause of death.

In this study, the youngest age group (25-44 years) demonstrated the largest increase in AUD mortality (40.47% in 2020 vs 33.95% in 2021) across all age groups. The increase was similar for both sexes (approximately 24.65% for women and men in 2020 vs 20.08% and 22.41% in 2021, respectively). Less than 10.00% of excess deaths overall and among all subgroups were attributable to COVID-19 infection (Figure).

## Discussion

In this cross-sectional study, we used data from 2012 to 2019 to project 2020 and 2021 mortality rates and found that AUD-related mortality rates increased among all ages and sexes during the pandemic. Younger persons, particularly those aged 25 to 44 years, had the steepest upward trend. The small proportion of COVID-19–related deaths suggests that excess deaths were more likely attributable to indirect effects of the pandemic such as stay-at-home policies and reduced medical and social resources for patients with AUD.

Our study is limited by potential misclassification bias in death certificate data. However, AUD is typically underrecognized, and we would expect such factors to result in lower observed death rates. Our findings suggest that the pandemic may have had a disproportionate association with AUD-related deaths and subgroups with high vulnerability and that tailored strategies are needed for AUD prevention and intervention to combat this public health crisis.
